# New Insights into IDO Biology in Bacterial and Viral Infections

**DOI:** 10.3389/fimmu.2014.00384

**Published:** 2014-08-11

**Authors:** Susanne V. Schmidt, Joachim L. Schultze

**Affiliations:** ^1^Genomics and Immunoregulation, LIMES-Institute, University of Bonn, Bonn, Germany

**Keywords:** IDO, viral infection, bacterial infection, depressive disorders, kyn metabolites

## Abstract

Initially, indoleamine-2,3-dioxygenase (IDO) has been introduced as a bactericidal effector mechanism and has been linked to T-cell immunosuppression and tolerance. In recent years, evidence has been accumulated that IDO also plays an important role during viral infections including HIV, influenza, and hepatitis B and C. Moreover, novel aspects about the role of IDO in bacterial infections and sepsis have been revealed. Here, we review these recent findings highlighting the central role of IDO and tryptophan metabolism in many major human infections. Moreover, we also shed light on issues concerning human-specific and mouse-specific host–pathogen interactions that need to be considered when studying the biology of IDO in the context of infections.

## Introduction

Indoleamine-2,3-dioxygenase (IDO) is an intracellular, non-secreted enzyme, which catabolizes the production of kynurenine (Kyn) derivates from tryptophan (Trp). Anti-proliferative features of IDO on bacteria, protozoa, and tumor cells have been first described in 1984 by Pfefferkorn (1) as well as Taylor and Feng (2). Induction of IDO in cells of the immune system by IFNγ was introduced for the first time in the late 1980s (3, 4). Today, IDO is thought to be part of a fast local immune regulatory mechanism called “metabolic immune regulation” to protect the host from over-reactive immune reactions via induction of systemic immune tolerance [reviewed elsewhere (5)]. It participates in a broad spectrum of immune responses during chronic infections, immune-escape of cancer cells, tissue inflammation, transplantation, and maternal tolerance toward the fetus and autoimmunity (6). Interestingly, accumulating evidence also connects enhanced Trp metabolism to mental disorders based on serotonin starvation.

Exogenous inflammatory stimuli induce the expression of IDO in antigen-presenting cells (APCs), such as dendritic cells (DC) (7), macrophages (4), and B-cells. Gene expression of IDO was found to be regulated by interferon-α (IFNα) and interferon γ (IFNγ) and also TNFα and prostaglandins (8, 9). As mode of action for IDO, ${\text{O}}_{2}^{▪}$-radical scavenging (2, 10) and later suppression of T-cell responses (11, 12) were discussed. Since many microbial organisms rely on the essential amino acid Trp, its degradation by IDO-expressing cells of the innate immune system was favored as the major IDO-mediated mechanism against infections (13). In infectious disease states, IDO has been shown to exert pleiotropic effects, even with opposing outcomes. On the one hand, IDO directly suppresses the replication of certain parasites and bacteria (1, 14–16), or at least prevents viral spread (17–20), on the other hand, it also acts on host cells to suppress immune reactions thereby promoting infectious diseases (21, 22). Besides Trp depletion, production of Trp metabolites with bactericidal activity, like Kyn, were identified in human macrophages upon infection with diverse bacteria species as another defense mechanism mediated by IDO (23). IFNγ induced Trp degradation leading to anti-toxoplasmosis activity in infected human fibroblasts was first described by Pfefferkorn in 1984 (1). Only a few years later, several studies linked this effect to enhanced IDO activity against pathogens like *Toxoplasma gondii*, certain *Chlamydia psittaci* strains and *Leishmania donovani* (14–16). In these initial studies, IDO-expressing immune cells were described as macrophages. A contribution of IDO in containment of viral infections was suggested by *in vitro* experiments demonstrating that the inhibition of human cytomegalovirus (CMV) replication was induced by IFNγ and IFNβ (18). This virostatic effect could be reverted by addition of exogenous Trp indicating an involvement of IDO (17, 19, 20). Interestingly, the activity of inducible nitric oxide synthetase (iNOS) was suggested to be able to substitute for the IDO-mediated anti-viral mechanism (18, 24). Since then, it was demonstrated that other viruses, such as herpes simplex virus type 2 (HSV-2) (17), measles virus (19), and vaccinia virus (20), are sensible to IDO-induced Trp depletion.

Apparently, pathogens are able to highjack the immunosuppressive effects of IDO and make use of them to facilitate their own life cycle. For instance, uropathogenic *Escherichia coli* (UPEC) induce IDO in epithelial cells of the urinary tract and in polymorphonuclear leukocytes (21). The dampened immune response upon IDO induction enables a successful colonization of urinary epithelium by UPEC. In addition, viruses like human immunodeficiency virus 1 (HIV) use the immunosuppressive activity of IDO to drive HIV infection into the chronic phase (25). In the following chapters, we will focus on new insights into the role of IDO and Kyn derivates in major viral and bacterial infections in mice and men.

## Role of IDO in Viral Infections

### Role of IDO in HIV infection

**Figure 1:**
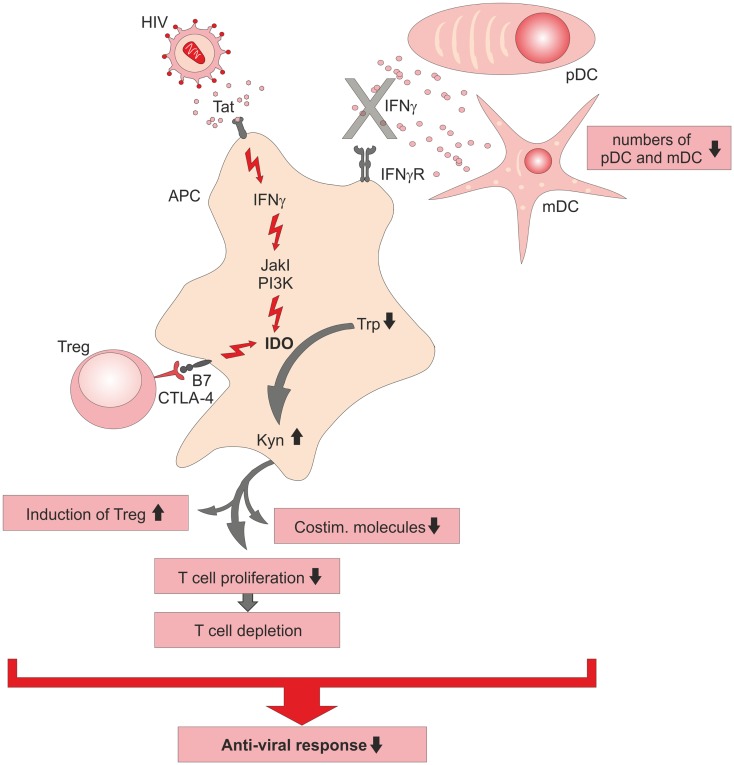
**Schematic summary of immunosuppressive functions of indoleamine-2,3-dioxygenase (IDO) during HIV infection**. Direct induction of IDO in antigen-presenting cells (APC) by viral Tat protein is established via an intracellular signaling cascade including kinases (JakI, PI3K) or CTLA-4-B7 interaction on regulatory T (Treg)-cells with B7 co-receptor on the APC, which leads in consequence to a breakdown of tryptophan (Trp) into kynurenine (Kyn). Diminished anti-viral immune responses during chronic HIV infection is caused by an impaired T-cell response, the lack of potent IFNγ secreting DC, and the induction of immunosuppressive IDO^+^ APC. pDC, plasmacytoid DC; mDC, myeloid DC.

Infection with HIV causes a severe impairment of T-cell responses by loss of proliferative capacity of T-cells accompanied by a depletion of functionally competent CD4^+^ T helper cells and by induction of regulatory T-cells (Treg) during the chronic phase of HIV infection (Figure 1). The exact T-cell impairing mechanism is still not completely understood, but inhibitory molecules on T-cell function have been investigated intensely [reviewed elsewhere (26)]. Elevated serum levels of IFNγ (27, 28) and Kyn (29) in HIV patients pointed toward a participation of IDO in suppression of T-cell function, yet molecular mechanisms were unknown. Further support came from increased IDO mRNA levels measured in peripheral blood mononuclear cells (PBMCs) of HIV-infected patients (30). *In vitro* infection of PBMC led to the secretion of IFNα and IFNβ by plasmacytoid dendritic cells (pDC) (31). While both CD4^+^ and CD8^+^ T-cells expressed the activation markers CD69 and CD38, they failed to proliferate and were insensitive to T-cell receptor stimulation, a status described as division arrest anergy (32). While CD4^+^ T-cells were arrested in G1/S phase, CD8^+^ T-cells downregulated the costimulatory receptor CD28. When the enzymatic activity of IDO was inhibited by 1-methyl tryptophan (1-MT), CD4^+^ and CD8^+^ T-cells regained their ability to proliferate (30, 31). In monocyte-derived DC (moDC), the N-terminal domain of HIV-1 transactivator regulatory protein (Tat) induced IFNγ and IDO expression and therefore further led to a suppression of T-cell proliferation. Here, 1-MT was also able to reconstitute T-cell proliferation (33). IDO expression was initially induced by Tat and followed by the induction of IFNγ leading to a feed forward loop. Interestingly, IFNγ signaling pathways leading to IDO expression could be blocked by JAKs and PI3K inhibitors but Tat-induced IDO expression could not be inhibited, suggesting a novel so far not characterized mechanism of IDO induction by Tat proteins in HIV infection (33).

In simian immunodeficiency virus (SIV)-infected macaques, treatment with a combination of antiretroviral therapy (ART) and 1-MT successfully diminished viral loads in plasma and lymph nodes and restored Trp levels but did not reduce Kyn (34). It is worth to mention that 1-MT alone was not able to restrain viremia in this animal model. Probably, IDO was only partially inhibited since reconstituted Trp levels were accompanied with elevated Kyn levels in sera of treated animals. Further, a compensatory counterregulatory mechanism for 1-MT was suggested due to increased IDO and TGFβ production in lymph nodes of treated animals. Decreased numbers of CD4^+^ T-cells during the course of HIV infection are accompanied by a loss of type I IFN producing cells, like pDC (35, 36). One reason for the low pDC numbers might be their redistribution to peripheral lymph nodes, as observed in the acute phase of SIV-infected macaques (36). Also, numbers of myeloid DC (mDC) are diminished in blood of HIV patients during primary infection (37). Yet, little is known about IDO expression in HIV-infected mDC. To investigate, if HIV alters the function of infected mDC, PBMC-derived DC were transfected with a HIV containing vector construct as a model system (38). This resulted in the induction of IDO in immature and matured DC, accompanied by increased levels of Kyn. In addition, elevated levels of TNFα and IFNγ were secreted by these DC with mature DC secreting the highest amounts. Further, HIV-transduced mature DC induced only modest T-cell proliferation in mixed-lymphocyte reactions, which might be due to IDO activity depleting Trp, a necessary molecule for T-cell function. The addition of 1-MT restored the immunostimulatory capacity of these DC, suggesting a central role of IDO in the suppression induced by HIV-infected DC.

Another mechanism of IDO expression in APCs is mediated by regulatory T-cells (Treg) (39, 40). In HIV patients, an elevated enzymatic activity of IDO in APCs was associated with a reduced anti-viral T-cell response [reviewed elsewhere (41)], while depletion of Treg cells reconstituted anti-HIV immune responses (42). Similarly, in SIV-infected macaques, the expression of the Treg markers CTLA-4 and FoxP3 was increased in T-cells of mesenteric lymph nodes, spleen, and colon, organs with high viral load (43). Simultaneously, IDO expression in spleen and gut-associated lymphoid tissues was suggested to support immunological suppression in favor of viral replication. Therefore, therapeutic targeting of Treg in HIV patients thereby reducing IDO expression in APCs and subsequently immunosuppression seemed promising. However, when blocking CTLA-4 signaling in SIV-infected macaques, an unexpected increase in IDO expression and Kyn levels was observed (44). Moreover, even under conditions of increased T-cell activation due to loss of the regulatory CTLA-4 signaling, viral replication was still promoted. Increased IDO levels were suggested to be a consequence of viral replication. In another attempt to refine ART by combining CTLA-4-blockade and 1-MT treatment, severe side effects causing acute pancreatitis with massive lymphocyte infiltration into the pancreas and loss of Langerhans islets were induced (45). Moreover, all tested animals developed diabetes and hyperglycemic coma while SIV-specific responses were not observed. These results clearly illustrate that the reversal of immunosuppression by targeting CTLA-4 in chronic viral infection is not a promising approach.

The influence of IDO expression on viral loads in mice infected with the retroviral leukemia virus LP-BM5 is controversially discussed. In an earlier study, higher numbers of pDC in IDO KO mice correlated with increased levels of type I IFN and reduced viral load (46). However, in a more recent study using the same model, IDO had no impact on disease progression (47). Viral loads of IDO KO mice were comparable to those of wild-type B6 mice and both IDO KO and WT mice showed decreased responsiveness to B-cell and T-cell mitogens. One might postulate that an important denominator of efficacy targeting IDO might simply be the time when IDO is induced during the course of the infection. Clearly, more work is necessary to determine the role of IDO in murine retroviral infections.

**Figure 2:**
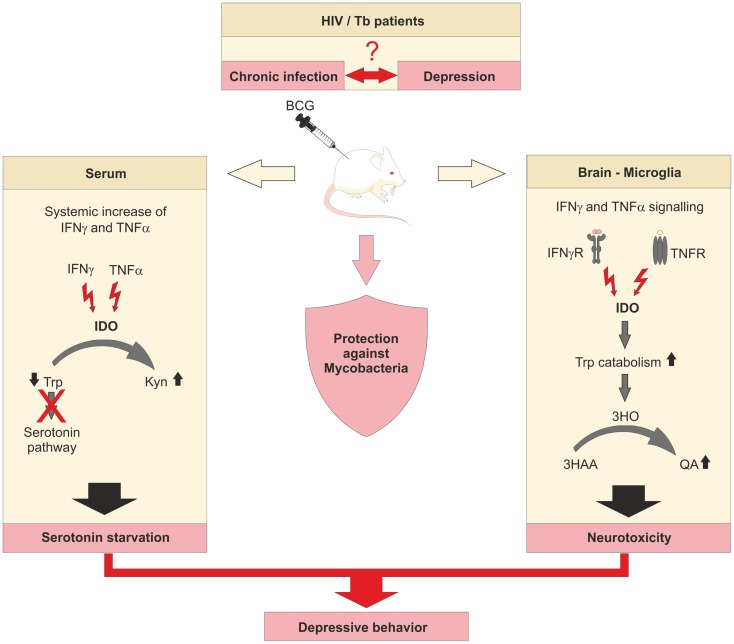
**IDO links chronic viral and bacterial infections to cases of depression**. Underlying mechanisms of the connection between chronic infection with viruses or bacteria and the onset of mental disorders in men are unknown. Insufficient levels of serotonin in chronically infected and depressive patients are thought to be the consequence of constitutively elevated levels of IDO. Murine models of chronic infections are established to decipher the involvement of IDO in the development of depressive-like behavior.

An interesting link between chronic inflammatory diseases and neurological disorders has been recently made with IDO being involved. Patients suffering from chronic inflammatory diseases show often signs of depressive mood behavior (Figure 2). In patients with chronic HIV infection, the elevated Trp catabolism maintained by IDO expression is associated with reduced levels of free serum Trp (48, 49). This correlates with a reduction of serotonin (5-HT) and serotonin transporter (5-HTT) expression, as well as an accumulation of neurotoxic Trp metabolites (50). Especially, Kyn and quinolinic acid (QA) can be detected in cerebrospinal fluids of HIV patients and are linked to the development of neuropsychiatric disorders, as part of the Neuro-AIDS complex of symptoms (50). HIV-1 associated dementia (HAD) is correlated to IDO and Kyn induced by Tat of HIV-1 clade B in human primary astrocytes (51) while Tat of HIV-1 clade C does not induce IDO activity in human primary astrocytes and is not associated with HAD. Further evidence for the role of Tat for IDO expression in the brain came from experiments injecting Tat protein intracerebroventricularly into different mice strains. Further, when injecting Tat, induction of IDO and several other pro-inflammatory cytokines in the brain was associated with reduced mobility and depressive-like behavior (52), demonstrating the important role of IDO in the pathophysiology of HIV infection. Blockade of IDO or upstream events of IDO induction in chronic infection might be a novel approach to treat chronic HIV infections.

### Role of IDO in influenza infection

So far, research of IDO function has focused mainly on murine influenza infection models. Infection with murine influenza virus PR8 has been shown to induce IDO expression in mouse lung tissue (53, 54) and lung-associated lymph nodes (54). IDO activity increased during influenza infection and peak expression correlated with increased lymphocyte numbers in the respiratory tract, albeit the study did not discriminate between T-cell and B-cell subpopulations (53). In another study, inhibition of IDO by 1-MT treatment in influenza-infected mice led to increased numbers of virus-specific memory CD8^+^ T-cells and functionally activated effector CD4^+^ T-cells (55). In a follow-up study, 1-MT-treatment improved memory T-cell responses correlated with increased secretion of IFNγ by CD4^+^ and CD8^+^ T-cells, accelerated Th1 responses, and a broader virus- and epitope-specific repertoire of CD8^+^ T-cells (56). Besides, 1-MT treatment led to improved repair of lung tissue. These results indicate that inhibition of IDO might improve flu vaccine activity, might aid in heterosubtypic immunity and support a faster recovery. This view is also supported by data reporting reduced morbidity rates in IDO KO mice when challenged with RP8 or X31 influenza viruses (54). Especially, IDO deficiency led to enhanced development of memory T-cells, which protected mice from lethal virus infection. Further, it was demonstrated that non-hematopoietic cells from lung-draining lymph nodes were major IDO producing cells in response to X31-induced IFNγ secretion. As a feed forward loop, IFN type I and II induced subsequently IDO activity in hematopoietic cells. As a next step, it will be important to translate these findings to human influenza infection and design clinical studies that would allow testing IDO blockade in context of infection and/or vaccination.

### Role of IDO in HBV and HCV chronic infection

Indoleamine-2,3-dioxygenase has also been linked to chronic infection with hepatitis B virus (HBV) and hepatitis C virus (HCV) (57). Despite an appropriate T-cell response during the acute phase of HCV infection and subsequent viral clearance, T-cell responses in the chronic phase are weak [summarized by Hiroishi et al. (58)]. A massive amount of HCV-specific CD8 T-cells are recruited to the liver; however, recognition of viral epitopes is barely present. Similarly, in chronic HBV infection, cytotoxic T lymphocytes (CTLs) show only a weak response against the HBV surface antigen (HBsAg). While the molecular mechanisms of the tolerogenic state in chronic HBV and HCV infection are not completely understood, an increased IDO expression in the liver of patients with chronic HBV or HCV infection has been observed (57). Moreover, high systemic Kyn/Trp ratios in chronically infected patients indicate increased IDO activation. In a recent study including 176 patients suffering from chronic HCV infection and 37 healthy controls, it could be shown that Kyn levels correlated with advanced liver inflammation and fibrosis (59). Furthermore, monocytes isolated from PBMCs of HCV patients differentiated into IDO^+^ DC were more potent in inducing Treg cells when activated with LPS or IFNγ than those of the control group. Along these lines, *in vitro* stimulation of the HCV-infected hepatocellular carcinoma cell line Huh7 with IFNγ led to an induction of IDO, yet HCV replication was not altered by IDO activity (57).

Yet another mechanism for induction of IDO expression was demonstrated in a murine hepatitis model. Treatment with α-galactosylceramide (α-GalCer), a specific agonist for natural killer (NK) cells was able to induce IDO (60). It was speculated that IDO suppresses an overactive immune response triggered by TNFα-producing NK cells and macrophages infiltrating the liver. Following the idea of re-establishing immunocompetent CTLs, wild-type and IDO KO mice were immunized with a combination of α-GalCer and HBsAg (61). Upon immunization, expression of the cytokines IL-2 and IL-12b were only increased in IDO KO mice leading to the induction of HBsAg-specific CTLs. Major IDO-expressing cells were CD11b^+^ Ly6G^+^ myeloid-derived suppressor cells (MDSCs) from spleen, which increased in numbers after immunization. They directly inhibited the proliferation of HBsAg-specific CTLs. To assess the role of genes induced by IFNα and/or IFNγ, Mao et al. co-transfected HepG2 cells with the HBV core promoter and 37 different expression plasmids for IFNα- and/or IFNγ-induced genes (62). Only IDO, APOBEC3G, PKR, and ISG20 reduced HBV DNA levels of more than 60%. IDO was considered as the major mediator of the IFNγ-induced anti-viral response, since it mediated Trp depletion followed by suppression of HBV replication.

### Role of IDO in other chronic viral infections

Indoleamine-2,3-dioxygenase might also play a role in several other viral infections. While infection with Epstein–Barr virus (EBV) normally causes a self-limited polyclonal lymphoproliferation, it was shown recently that EBV-transformed B-cells express elevated levels of IDO causing Trp degradation to Kyn, which – in turn – suppressed the expression of the activating receptor NK group 2, member D (NKG2D) receptor on the surface of NK cells (63). This might be important since NK cells have been suggested to control the proliferation of EBV-infected B-cells in the acute phase. In the same report, it was shown that IL18 suppressed the effect of Kyn on NKG2D expression. It might be speculated that the suppression of NK cell activation by IDO-expressing EBV-infected B-cells serves as an escape strategy of the virus. Recently, it was shown that *in vitro* generated macrophages expressed IDO after infection with EBV and displayed T-cell suppressive activities (64). IDO expression induced by TNFα and IL6 signaling was mediated by NFκB and the MAP kinase signaling cascade. Both factors were able to further increase IDO expression, thereby suppressing the proliferative capacity of CD4^+^ and CD8^+^ T-cells as well as dampening their cytolytic activity.

Additional evidence for an important role for IDO in viral infection was provided in studies of human papilloma viruses (HPV). Mucosa-tropic HPV are tumorigenic viruses causing genital cancer, e.g., cervical cancer by inducing epithelial hyperplasia (65). Immunotherapy against the tumor is often inefficient because of the existence of a local immunosuppressive tumor milieu with impaired tumor cell antigen presentation and resistance of tumor cells against effector mechanisms of T-cells. The immunosuppressive milieu might be created during the stage of cervical intraepithelial neoplasia (CIN), since numbers of IDO, IFNγ, IL10, and FoxP3 expressing cells are elevated in CIN compared to normal cervical tissue (66). When HPV16 E7, an envelope- and oncoprotein of HPV was expressed under the keratin-14 promoter (K14E7) in a skin grafting model, graft rejection was prevented suggesting that HPV16E7 induced an immunotolerogenic environment (67). As previously shown, tolerance against E7-expressing skin grafts is based on the induction of IFNγ producing natural killer T (NKT)-cells, which not only reduce the capacity of CD11c^+^ DC to cross-present antigens to CD8 T-cells (68) but also seem to induce IDO expression. In fact, IDO seems to have a pivotal role in K14E7 graft tolerance, since IDO inhibition by 1-MT leads to rejection of K14E7 skin grafts (67). Furthermore, it was observed that skin grafts recruit higher numbers of DC, which expressed elevated levels of IFNγ receptor (IFNγR). Especially, dermal Langerin^+^ (CD207^+^) DC expressed IDO and aided in the recruitment of further DC to the side of transplantation. Clearly, this murine model strongly suggests an important participation of IDO in HPV evasion from host immunity.

Overall, IDO seems to play an important role in chronic viral infections mainly by contributing to the establishment of an immunotolerogenic microenvironment. New strategies to target IDO itself or upstream mechanisms inducing IDO might help in the development of therapeutic drugs for patients with chronic viral infections.

## Novel Aspects of IDO in Major Bacterial Infections

### Role of IDO in mycobacteria infections

**Figure 3:**
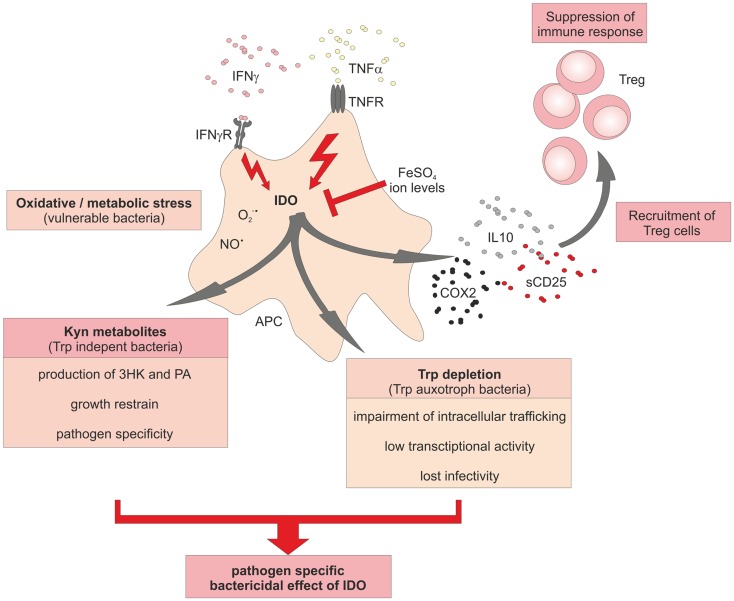
**Schematic overview of the central role of IDO in immune responses to bacterial infections**. Activation of IDO activity in bacteria-infected cells induces a potent bactericidal growth restrain to fight against spreading of the infection. Besides the induction of reactive oxygen species (ROS) and nitric oxide radicals, activity of IDO aids in Trp degradation to starve Trp auxotroph bacteria. A further species-specific bactericidal effect of increased IDO activation is the production of toxic Kyn metabolites. Recruitment of Treg cells and increasing levels of free ions might help as negative feedback loop to terminate inflammatory responses.

Tuberculosis (Tb), caused by *mycobacteria*, is one of the major human infectious diseases. We and others have linked IDO expression to this disease (69–72). In Tb patients elevated levels of anti-inflammatory molecules in the sputum, amongst them IDO have been detected (70). These immune suppressive mediators, also including IL10, TGFßRII, and IL1 receptor antagonist (IL1Rn), have been suggested as biomarkers for Tb. It was assumed that all these inhibitory molecules dampen Th1 responses in the lung thereby contributing to immune-escape of the bacteria. While the direct molecular mechanism of IDO induction in Tb patients is unresolved, it is clear that IFNγ and TNFα play pivotal roles in the containment of *Mycobacterium tuberculosis* (*M. tuberculosis*) in humans and in mice (Figure 3). Deficiency of IFNγ or TNFα expression, or the lack of the respective receptors cause severe courses of Tb in mice (73–77). From *in vitro* studies, there is evidence that TNFα might play a role during the acute and chronic phase of Tb (9, 76). Moreover, treatment of patients with anti-TNFα antibodies can lead to exacerbation of Tb and the induction of Tb sepsis (78). *In situ* studies clearly demonstrated that granuloma formation after *M. tuberculosis* infection in humans is associated with high expression of IDO in cells of the center and the ring wall structure surrounding the center of the granuloma (9, 71, 79). Predominant IDO-expressing cells were identified as CD68^+^ macrophages and in fewer numbers as CD11c^+^ S100^+^ DC. Both IDO-expressing myeloid cell populations were surrounded by CD3^+^ T-cells. The induction of a tolerogenic milieu including the recruitment of FoxP3^+^ regulatory T-cells by IDO-expressing cells together with depletion of Trp most likely aids in the restriction of bacterial spread. IDO seemed to be absent in biopsies of *Mycobacterium leprae* (*M. leprae*) infected skin, suggesting that IDO is not necessarily involved in all granulomatous diseases. However, there is still controversy about this, since a recent report demonstrated expression of IDO in macrophages of leprotic skin lesions (80).

Further controversy about the role of IDO in Tb comes from a recent report demonstrating no essential role for IDO in a murine model of Tb (81). Wild-type as well as IDO KO mice showed comparable bacterial burden, T-cell responses, and survival rates, leading to the conclusion that IDO activity is not required for the control of *M. tuberculosis*. The authors present no evidence IDO is indeed expressed in the myeloid cell compartment, like in human disease, indicating that this mouse model might not reflect the human situation. In contrast, another report demonstrates a direct connection between IFNγ and IDO expression in non-hematopoietic cells, during the chronic phase of murine *M. tuberculosis* infection (82). In this model, IDO was expressed in endothelial and epithelial cells, and IFN receptor deficiency reduced the levels of expression of IDO in these cells thereby leading to an impaired long-term control of *M. tuberculosis* (82). However, it was also not shown to which extent IDO activity directly controls *M. tuberculosis*.

There have been numerous studies during the last 20 years demonstrating a correlation between Tb and depressive disorders in patients (Figure 2) (83–86), but many of the underlying molecular mechanisms remain unclear. To shed some light on the mechanism, O’Connor et al. utilized a BCG vaccination model (87). Mice that showed a depressive-like behavior also represented increased expression of IFNγ, TNFα, and IDO. When studying IFNγ-R deficient mice, many inflammatory mediators were still elevated while the expression of TNFα was attenuated and IFNγ and IDO were absent in lung or brain tissue. Furthermore, the lack of IDO activity in IFNγ-R deficient mice resulted in diminished plasma ratios of Kyn/Trp. The authors assumed a synergistic effect of IDO and TNFα on microglia of BCG-treated mice since pretreatment of mice with a TNFα antagonist was able to attenuate TNFα expression and to abrogate depressive-like behavior. Further studies need to clarify whether TNFα is actually upstream of IDO and blockade of TNFα can reduce IDO expression and function. Downstream of IDO, BCG also increased the expression of 3-hydroxyanthranilic acid oxygenase (3-HAO), which participates in the generation of the neurotoxic Kyn metabolite QA (88). Application of 1-MT also prevented the development of depressive-like behavior after BCG inoculation in mice, suggesting that elevated IDO, like TNFα, contributes to the onset of depressive disorders during chronic inflammation caused by *mycobacteria*.

### Role of IDO in *Chlamydia* infections

Indoleamine-2,3-dioxygenase was also shown to be important in the defense against the ubiquitous intracellular bacterium *Chlamydia pneumoniae* (*C. pneumoniae*), which causes respiratory tract infections and is associated with chronic diseases like asthma. In general, the life cycle of *Chlamydia* species can be divided into two phases: (1) a stage of infectious but metabolically inactive form directly after uptake of the bacteria into the host cell, and (2) a stage of differentiation and multiplication enabled by an active metabolism. It can be assumed that immune responses of host cells might differ according to the life cycle of *Chlamydia*. Njau et al. recently demonstrated that *C. pneumoniae* infection of human moDC-induced IDO expression in a TNFα-dependent manner and IDO was sufficient to restrain bacterial growth (89). TNFα-dependent bactericidal effects on *C. pneumoniae* were abrogated after supplementation of Trp. Since *C. pneumoniae* is Trp auxotroph, the authors concluded from these experiments that Trp depletion is detrimental for *C. pneumonia* growth. In *C. pneumoniae*-infected THP-1 cells, an induction of IDO, TNFα, and neopterin could be demonstrated (90). However, IDO activation in combination with increased Trp degradation as well as IFNγ treatment had no effect on numbers or growth of *C. pneumoniae* in THP-1 cells. This observation was explained by the ability of *C. pneumoniae* to survive even under conditions of low Trp concentrations, going into a latent state without proliferation and differentiation. An interesting aspect of this study was the comparison of chlamydia infection in THP-1 cells and human endothelial cells. While IDO was already induced in monocytic cells upon infection and was further elevated by IFNγ, infected and non-infected endothelial cells required IFNγ stimulation to induce IDO expression. Only after IFNγ-treatment, suppression of proliferation of *C. pneumoniae* in endothelial cells was observed. The authors also argued that the differences between the two cell types might also reflect different stages of the *C. pneumoniae* infectious cycle. The bacteria might use immune competent monocytes as transportation vessels for systemic dissemination, while endothelial cells might serve as habitats for differentiation and proliferation, especially under iron rich conditions.

An interesting link between IDO and iron metabolism has been suggested by Krausse-Opatz and colleagues (91). In a hepatic cell line infected with *Chlamydia trachomatis* (*C. trachomatis*), they could demonstrate that increased intracellular levels of ferrous iron FeSO_4_ attenuated IFNγ-induced IDO expression leading to increased infectious yields. Similarly, the human monocytic cell line THP-1 has been shown to be sensitive to low iron concentrations, which showed an inhibitory effect on IFNγ signaling resulting in decreased Trp metabolism (92). This observation led to the hypothesis that immune cells retain iron during inflammatory diseases to enable efficient IFNγ driven immune responses. On the other hand, reduction of iron levels by deferoxamine did not reconstitute IDO activity, but still suppressed bacterial growth (90, 91). Leonhardt et al. addressed whether IDO-mediated Trp depletion is responsible for suppression of bacterial growth of *C. trachomatis* (93). They used HeLa cells, an immortalized cervical carcinoma cell line, as the model for infection. Interestingly, the lack of available Trp in IDO competent HeLa cells led to an impairment of intracellular bacteria trafficking toward the perinuclear microtubule-organizing center after entrance into the host cell. Bacteria displayed low transcriptional activity, lost their infectivity, and remained scattered in the periphery of the HeLa cells. Since most of the recent work concerning the influence of iron ions has been performed in cell line models, it remains an unanswered question, whether these observations will hold true, once primary human cells are used for analysis.

### Role of IDO in community-acquired pneumonia and sepsis

Another area of great interest during the last years has been the function of IDO during community-acquired pneumonia (CAP) and sepsis. Already in 2005, increased Kyn levels in trauma patients with bacteremic sepsis, respiratory distress syndrome, or multi organ dysfunction/failure pointed toward a role for IDO in sepsis (94). In a larger cohort-study, Suzuki and colleagues investigated 129 patients and 64 healthy controls and revealed increased Kyn/Trp ratios as prognostic marker for severity and morbidity of CAP caused by multiple pathogens (95). Along these lines, several independent studies reported increased IDO activity as predictor of severity and mortality of sepsis (96–98). Elevated Kyn/Trp ratios as indicator of IDO activity in plasma of sepsis patients also correlated with elevated levels of IFNγ and IL10, which were also shown to further trigger IDO activity (96). Furthermore, these patients show reduced CD4^+^ and CD8^+^ T-cell counts similarly pointing to an overall impairment of immune functions during sepsis. There was also an inverse correlation between elevated Kyn/Trp ratios and NO-dependent microvascular reactivity as a surrogate marker for endothelial cell function, as mean arterial or diastolic blood pressure were reduced. Direct measurement of IDO activity in patients with sepsis or septic shock showed a gradual increase of IDO activity with severity of sepsis, which was directly associated with mortality (97). The major sources of IDO were circulating CD14^+^ monocytes, which were increased both absolutely and relatively compared to other white blood cells during sepsis. Interestingly, *ex vivo* stimulation of monocytes from septic patients with IFNγ led to the induction of functional IDO and was found to be independent of NFκB signaling while other TLR agonists like LPS-induced IDO expression in an NFκB dependent fashion (97).

Along these lines, hints for a novel IDO-inducing mechanism were found in a murine mouse model in which the induction of IDO in context of sepsis was investigated. Mice which are deficient in serine/threonine kinase, general control non-derepressible 2 (GCN2) gene were protected from endotoxic shock (99). This was associated with a rapid induction of IDO in spleen and an increased Kyn/Trp ratio in serum suggesting that elevated IDO levels in sepsis might actually be beneficial. However, this does not seem to be necessarily the case since IDO deficient mice also are resistant against LPS-induced septic shock (100). Similarly, inhibition of IDO activity by 1-MT during endotoxin shock *in vivo* was associated with increased survival (100). Whether these results can be translated to humans has to be proven. In this context, it is interesting to mention that application of GMCSF in sepsis patients was associated with suppression of IDO activity and reduction of free Kyn metabolites (101). Experimental proof for concomitant improvement of antibacterial defense has to be further investigated.

Overall, IDO also seems to play a role during clinically relevant infections such as CAP and sepsis. Whether IDO might become a therapeutic target in these patients is still to be investigated.

### Role of IDO in *Listeria* infections

During the last years, we have defined the role of IDO in human listeriosis (8, 9, 23, 69, 79, 93, 102). Listeriosis is a foodborne disease caused by oral infection with *Listeria monocytogenes* (*L. monocytogenes*). Newborn infants are prone to infections due to incompletely developed cell-mediated immunity. Early on, we could demonstrate that induction of IDO in myeloid cells after *L. monocytogenes* infection is TNFα dependent (9). Chronic listeriosis is characterized by the development of granulomatous structures encapsulating bacteria-infected cells to restrain bacterial spreading. IDO-expressing CD68^+^ macrophages and S100^+^CD11c^+^ DC, but not T- or B-cells, are part of the ring wall structure in these granuloma. When analyzing the effect of *L. monocytogenes* infection on DC function, we observed an inhibitory effect of these DC on T-cell proliferation and cytokine expression. Furthermore, we demonstrated that the inhibitory effect is mediated by IDO and the TNFα-dependent production of suppressive molecules like IL10, COX2, and soluble CD25 (69). Initial experiments addressing the IDO-mediated inhibitory effects on T-cells suggested that IDO-mediated Trp depletion and also the production of toxic metabolites were responsible for the observed loss of T-cell proliferation and cytokine production (69, 102). More recently, we were interested to understand whether bactericidal effects exerted by IDO-expressing human myeloid cells is mainly due to Trp depletion or toxic metabolites (23). Earlier work in mice and in human cells, as well as in other bacterial infections showed conflicting results concerning the role of these two bactericidal mechanisms. Using primary human macrophages and DCs, we could show unequivocally that it is not Trp depletion but rather Kyn metabolites that exert the bactericidal effect against *L. monocytogenes*. Especially, 3-hydroxy-kynurenine (3HK) was the most potent Kyn metabolite. Moreover, this bactericidal effect was also seen for other Listeria strains including *Listeria innocua*. It is important to note that these findings only reflect IDO biology in humans, since we could also clearly show that IDO is not induced in murine DC and macrophages after infection with *L. monocytogenes*. In contrast, murine *L. monocytogenes* infected myeloid cells produced iNOS instead of IDO. One explanation of the observed species-specific differences might be a differential expression of the iNOS cofactor tetrahydrobiopterin, which is expressed by murine primary macrophages as well as murine macrophage cell lines after activation with IFNγ and LPS (103, 104). In fact, murine IFNγ stimulated macrophages showed a strong correlation between tetrahydrobiopterin and NO levels (104). In contrast, tetrahydrobiopterin was shown to be expressed only at very low levels in human monocytes and macrophages (104, 105). A further explanation for this divergent usage of IDO and NO between mice and men came from a study, which revealed an inhibitory effect of NO on IDO in primary IFNγ-activated human peripheral mononuclear cells and macrophages (106). An interaction of NO with the heme iron at the active site of IDO preventing the conversion of ferric iron, necessary for IDO activity, has been proposed to be responsible for the inhibitory effect of NO on IDO (107). IDO inhibition might also rely on rapid removal of oxygen radicals by NO, which are required for IDO activity (108, 109). Such a clear-cut species-specific difference in usage of effector molecules as well as diverging susceptibilities against pathogens between mice and men are not too surprising since both species have evolved in different habitats, being exposed to completely different sets of pathogens. These observations further support the notion that one needs to be very careful when studying murine IDO in infection models and translating gained knowledge back to human diseases such as listeriosis or Tb.

## Summary/Concluding Remarks

Recent findings have clearly revealed that elevated IDO expression is a hallmark of major human viral infections including HIV, HBV, HCV, or influenza, and also major bacterial infections such as Tb, CAP, listeriosis, or sepsis. Besides Trp depletion, production of ROS or the modulation of Trp metabolism by iron ions via IFNγ have to be considered as part of a complex network participating in the fight against pathogens. An elevated Kyn/Trp ratio as a surrogate for IDO activity seems to be a hallmark for these infectious diseases and as demonstrated for sepsis patients seem to correlate with the severity of disease. So far, the major role of elevated IDO activity has not been definitely established. Both anti-pathogen and immunosuppressive mechanisms have been suggested. In this scenario, IDO activity might be a dual sword, fighting the pathogen directly by a metabolic mechanism, while keeping an overwhelming immune response in check. Whether this delicate balance between pathogen defense and host protection from lateral damage induced by IDO could be utilized therapeutically to further optimize the host response during infection remains to be seen. So far, it is not clear whether a further increase of IDO activity would indeed be beneficial. It might enhance the anti-pathogenic effect, but further elevating immunosuppression might actually be detrimental to the host during infection.

In fact, data from septic patients as well as studies from murine sepsis models utilizing LPS to induce septic shock would actually speak against enhancing IDO activity. On the contrary, in these models, depletion of IDO function is actually beneficial to the host. As exemplified above, interpreting the role of IDO for human Tb might be very misleading when based on murine models that do not reflect human disease properly. Moreover, we have clearly established that IDO is differentially regulated in murine and human myeloid cells in response to infection with *L. monocytogenes* and probably also other viral and bacterial infections. Particularly when studying the myeloid compartment, we strongly recommend to study regulation and function of IDO first in humans. If IDO is involved, the findings should be translated into the murine model. However, if there is no evidence for IDO regulation in the same cellular compartment in the murine infection model, then it will be necessary to either use other more informative animal models or to apply other approaches in the human setting. For future research, it will be extremely important to develop structured datasets that can be quickly interrogated to understand whether gene function observed in human disease is also apparent in major animal models, particularly the murine system. So far, such data are missing.

## Conflict of Interest Statement

The authors declare that the research was conducted in the absence of any commercial or financial relationships that could be construed as a potential conflict of interest.
